# Association between serum alkaline phosphatase and renal outcome in patients with type 2 diabetes mellitus

**DOI:** 10.1080/0886022X.2020.1804402

**Published:** 2020-08-12

**Authors:** Lijun Zhao, Lin Li, Honghong Ren, Yutong Zou, Rui Zhang, Shanshan Wang, Huan Xu, Jie Zhang, Fang Liu

**Affiliations:** a Division of Nephrology, West China Hospital of Sichuan University, Chengdu, Sichuan, China; b Division of Pathology, West China Hospital of Sichuan University, Chengdu, Sichuan, China; c Key Laboratory of Transplant Engineering and Immunology, Ministry of Health, Regenerative Medicine Research Center, Chengdu, Sichuan, China

**Keywords:** Alkaline phosphatase, diabetic nephropathy, proteinuria, fibrosis, end-stage renal disease, sFRP2-Wnt signaling

## Abstract

This retrospective study included 299 patients with type 2 diabetes mellitus and biopsy-confirmed diabetic nephropathy (DN) to investigate the prognostic value of alkaline phosphatase (ALP) for renal outcome. Cox proportional hazards models were used to estimate the hazard ratios (HRs) for the serum ALP level on renal outcome, which was defined as end-stage renal disease (ESRD) or a 50% decline in estimated glomerular filtration rate (eGFR) from baseline. The median baseline ALP was 80 IU/L with an interquartile range of 64–97 IU/L. Serum ALP was negatively associated with eGFR but positively associated with proteinuria and renal interstitial fibrosis. During a median follow-up period of 23 months, ESRD or a 50% declined in the eGFR occurred in 156 (52.2%) patients. The highest quartile of ALP was significantly associated with poor renal outcome, as defined above (HR 2.38, 95% confidence interval [CI] 1.09–5.17), when adjusted for sociodemographics, baseline eGFR, proteinuria, liver function parameters, parathyroid hormone levels, and renal pathological findings. Each standard deviation higher in the natural log-transformed ALP was associated with a 25% increased risk for poor renal outcome. Additionally, there was a graded increase in the risk for poor renal outcome with higher ALP in patients with nephrotic-range proteinuria. However, no significant associations were observed between serum ALP levels and renal outcome in patients with non-nephrotic-range proteinuria. In conclusion, an elevated ALP level was independently associated with poor renal outcome in patients with type 2 diabetes mellitus and nephrotic-range proteinuria after multivariate adjustment.

## 1. Introduction

The global pandemic of diabetes mellitus (DM) is perhaps the biggest epidemic in human history, with an estimated 463 million adults living with diabetes worldwide in 2019 [1]. Approximately 21% of patients with DM develop diabetic nephropathy (DN), a kidney-related complication of diabetes, which has become the leading cause of end-stage renal disease (ESRD) in China [2]. To date, the only widely used predictors of ESRD are clinical parameters such as estimated glomerular filtration rate (eGFR), albuminuria, and blood pressure [3–5]. A novel, noninvasive prognostic marker has yet to be discovered for DN.

Alkaline phosphatase (ALP) is present in a variety of tissues but it is especially abundant in the liver, bone, and kidney. Serum ALP has been widely considered a surrogate of mineral metabolism. Imbalances in bone and mineral metabolism are seen in almost all patients with DN or those receiving dialysis and are associated with a higher risk of adverse clinical outcomes [6,7]. Several cross-sectional studies have shown that a high level of circulating ALP is a good predictor of mortality or ESRD in patients with chronic kidney disease [8–12]. In diabetic patients, vascular calcification appears to be a strong independent predictor of cardiovascular mortality and kidney progression [13,14]. ALP has been shown to play a critical role in calcification; it has been extensively used as a marker for and exploited as a therapeutic target of vascular calcification [15]. A number of studies have revealed that ALP is correlated with glucose metabolism, insulin resistance, and metabolic syndrome due to its role as a hepatobiliary marker [16–18]. In a recent study, insulin stimulated the proliferation and differentiation of osteoblasts, and resulted in an increase in bone-specific ALP levels *in vitro* [19]. In patients with type 1 diabetes, increased serum levels of ALP were associated with glomerular hyperfiltration and kidney progression in the early stages of DN [20,21]. However, there was no data on the association between serum ALP and kidney progression in patients with type 2 diabetes (T2DM) and associated DN. Thus, this study was aimed to evaluate the relationship between ALP level and renal prognosis in patients with T2DM and biopsy-proven DN.

## 2. Materials and methods

### 2.1. Setting and population

**Figure 1.**
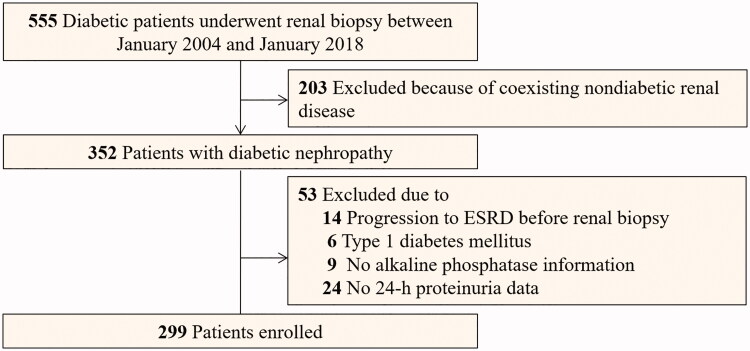
Flowcharts of participants in this study.

We conducted a retrospective study of patients with T2DM and DN who underwent percutaneous renal biopsy between 2004 and 2018 at the West China Hospital of Sichuan University. Renal biopsy was performed in T2DM patients with renal damage who lacked absolute contraindications [22–24]. The diagnosis of T2DM was made according to the American Diabetes Association criteria [25]. DN was diagnosed by at least two renal pathologists and/or nephrologists using the Renal Pathology Society (RPS) classification [26]. Adult patients who were followed up at our hospital for at least 1 year were eligible for the present study. The exclusion criteria were the presence of coexisting non-diabetic renal diseases such as IgA nephropathy; systemic diseases (e.g., antineutrophil cytoplasmic antibodies-associated vasculitis, anti-glomerular basement membrane disease, and lupus nephritis); progression to ESRD prior to renal biopsy; and type 1 diabetes mellitus. Patients lacking 24-h proteinuria or serum ALP information were also excluded from this study (Figure 1). Ultimately, 299 patients with T2DM and biopsy-confirmed DN were enrolled. All patients provided written informed consent, and the study was approved by the institutional review board of the West China Hospital of Sichuan University [Approval number: 2003 (1)].

### 2.2. Clinical information and pathological classification

Baseline complete clinical data at the time of renal biopsy were abstracted from electronic medical records. The eGFR was evaluated using the Chronic Kidney Disease Epidemiology Collaboration (CKD-EPI) formula [24]. Serum ALP was measured using a colorimetric assay in accordance with a standardized method (Roche Modular Analyzer) [12].

Renal biopsy samples were prepared for light microscopy, immunofluorescence, and electron microscopy using standard procedures at West China Hospital. The glomerular, interstitial, and vascular compartments were scored according to the RPS classification [26]. The glomerular classifications were categorized into five classes: class I: GBM thickening; class IIa: mild mesangial expansion; class IIb: severe mesangial expansion; class III: nodular sclerosis; and class IV: global glomerulosclerosis in >50% of glomeruli. IFTA was scored as follows: 0, absent; 1, <25%; 2, 25–50%; and 3, >50% of the total area. Interstitial inflammation was scored as follows: 0, absent; 1, inflammation only in relation to IFTA; and 2, inflammation in areas without IFTA. Arteriolar hyalinosis was scored as follows: 0, absent; 1, at least one area of arteriolar hyalinosis; and 2, more than one area of arteriolar hyalinosis. Arteriosclerosis was scored as follows: NA, absence of large vessels; 0, no intimal thickening; 1, intimal thickening less than the medial thickness; and 2, intimal thickening greater than the medial thickness [26,27]. Epithelial tubular degeneration was further assessed and determined as score 1, light changes exhibited swollen change; score 2, moderate changes, exhibited vacuolar degeneration; score 3, severe changes, exhibited brush border membrane shed and naked [28]. Renal specimens were examined by two nephropathologists (L.L. and H.X.) and any scoring discrepancies were resolved by discussion. These pathologists were blinded to the clinical data and renal outcome.

### 2.3. Renal outcome

Renal outcome was defined as the progression to ESRD or a 50% decline in the eGFR from baseline. The ESRD was indicated by an eGFR <15 mL/min/1.73 m^2^, or the commencement of renal replacement therapy [27,29]. All patients were followed until March 2019.

### 2.4. Statistical analysis

Continuous variables are expressed as means and standard deviations (SDs) if normally distributed, or as medians and interquartile ranges (IQRs) if not normally distributed. Categorical variables are expressed as counts and percentages. In patients with differing ALP level, differences in continuous variables were analyzed using one-way ANOVA, followed by the Bonferroni or Tukey methods for multiple comparisons, or the Kruskal–Wallis H test, as appropriate. Categorical variables were analyzed by Chi-square test or Fisher’s exact test [24].

Survival cures for four quartiles of serum ALP levels were obtained using the Kaplan–Meier method and log-rank tests. Univariate and multivariable Cox proportional hazards models were used to estimate the HRs for renal outcome [30]. No patients were lost to follow-up. Baseline serum calcium and phosphorus were missing for eight individuals. Differences in clinical parameters between patients with and without missing values were assessed first, to check whether the distribution of missing values was random. Multiple imputation methods were then used to derive multivariable models [31]. The proportional hazards assumption in Cox models was tested to check whether the datasets satisfied the inherent assumptions of Cox analysis. Then three multivariable Cox proportional hazard models [8] calculate HRs and 95% confidence intervals (CIs) for renal outcome. The first multivariable model included age, sex, baseline eGFR, 24-h proteinuria, gamma-glutamyl transferase (GGT), and total bilirubin. In the second multivariable model, the HRs were adjusted for the aforementioned factors plus renal pathological parameters that were *p < *0.05 in univariate models. The third multivariable model further adjusted for the baseline parathyroid hormone (PTH) and the usage of renin-angiotensin-aldosterone system inhibitors. The clinical covariates were selected as potential confounders because of their significance in univariate analysis or suggested in the literature to be associated with a higher risk of renal outcome [8,12,32]. Age and sex were chosen on the basis of biological plausibility. The quartiles of serum ALP levels as a categorical variable with the group quartile 1 (Q1) regarded as the reference, was used to explore the association between serum ALP and renal outcome. Serum ALP was also analyzed as a continuous variable with HRs calculated per SD increment of natural log-transformed ALP. The incremental prognostic value of adding serum ALP level in the model, *versus* a model that only contained renal functional parameters and pathological findings, was analyzed by calculating Harrell’s C-statistic, the likelihood ratio, and assessing the Akaike information criterion (AIC) [29]. Linear regression analysis was used to determine correlations between serum ALP level and clinical parameters. Cochran-Armitage trend test was further used to check the association between serum ALP quartiles and RPS glomerular classifications and IFTA scores.

Statistical analyses were performed using Stata version 14.0 (StataCorp LLC, College Station, TX, USA) or SAS version 9.4 (SAS Institute Inc, Cary, NC, USA). *p* < 0.05 was deemed to indicate statistical significance.

## 3. Results

### 3.1. Clinical and pathological characteristics of study patients

A summary of the demographics and baseline characteristics of the 299 patients enrolled in the study is shown in Table 1. The mean age of the participants was 51 years, and the group was composed of 94 women (31.4%) and 205 men (68.6%). At the time of biopsy, the median baseline eGFR was 59.8 mL/min/1.73 m^2^ and the median baseline 24-h proteinuria was 4.33 g/d. The median baseline ALP was 80 IU/L with an interquartile range of 64–97 IU/L. Patients were stratified by the quartiles of baseline serum ALP levels: Q1: ≤64 IU/L; Q2: 65–80 IU/L; Q3: 81–96 IU/L; Q4: ≥97 IU/L. Compared to patients in the Q1 group, patients in the Q2, Q3, or Q4 groups had higher glycated hemoglobin (HbA1c) and fasting plasma glucose. Patients in the Q4 group had lower eGFR, hemoglobin, albumin concentrations, and total bilirubin level, and heavier proteinuria than those in the other groups. Patients in the Q4 group used renin-angiotensin-aldosterone system inhibitors less frequently than those in the Q1, Q2, and Q3 groups. However, the body mass index, systolic/diastolic blood pressure, diabetes duration, direct bilirubin level, and serum PTH levels were distributed similarly among the four groups. There were no significant differences in the use of other medications among patients in the four groups.

**Table 1.** Clinical and pathologic features of patients with type 2 diabetes mellitus and diabetic nephropathy.

Characteristics	All	Serum ALP				*p* Value
		Q1 (*n* = 72)	Q2 (*n* = 78)	Q3 (*n* = 72)	Q4 (*n* = 77)	
	(*n* = 299)	≤64 IU/L	65–80 IU/L	81–96 IU/L	≥97 IU/L	
Age, mean (SD), years	51 (9)	51 (10)	50 (9)	52 (8)	51 (10)	0.80
Sex, Male, No. (%)	205 (68.6)	54 (75.0)	50 (64.1)	45 (62.5)	56 (72.7)	0.27
Ethnicity						0.31
Han, No. (%)	270 (90.3)	69 (95.8)	68 (87.2)	64 (88.9)	69 (89.6)	
Tibetan, No. (%)	29 (9.7)	3 (4.2)	10 (12.8)	8 (11.1)	8 (10.4)	
Smoking, No. (%)						0.99
Never smoking	161 (53.8)	39 (54.2)	42 (53.8)	39 (54.2)	41 (53.2)	
Ex smoking	44 (14.7)	9 (12.5)	13 (16.7)	10 (13.9)	12 (15.6)	
Current smoking	94 (31.4)	24 (33.3)	23 (29.5)	23 (31.9)	24 (31.2)	
History of DR, *n* (%)	155 (51.8)	35 (48.6)	35 (44.9)	40 (55.6)	45 (58.4)	0.31
History of Hypertension, *n* (%)	254 (84.9)	60 (83.3)	65 (83.3)	63 (87.5)	66 (85.7)	0.87
History of CVD, *n* (%)	54 (18.1)	11 (15.3)	10 (12.8)	14 (19.4)	19 (24.7)	0.24
BMI, mean (SD), kg/m^2^	25.5 (3.9)	25.9 (4)	26.1 (3.5)	25.7 (4.5)	24.1 (3.2)	0.06
SBP, mean (SD), mmHg	146.3 (23.6)	143.7 (23.7)	143 (24.2)	147 (22.2)	151.3 (23.8)	0.11
DBP, mean (SD), mmHg	86.9 (13.0)	84.5 (12.6)	85.9 (13.5)	87.5 (13.2)	89.5 (12.6)	0.11
MAP, mean (SD), mmHg	106.7 (14.9)	104.2 (14.5)	104.9 (15)	107.3 (14.5)	110.1 (14.9)	0.06
Duration of diabetes, median (IQR), months	84 (36–132)	96 (36–132)	84 (48–120)	78 (24–132)	96 (36–144)	0.93
HbA1c, median (IQR), %	7.0 (6.1–8.4)	6.7 (6.2–7.9)	7.7 (6.5–8.9)	7.1 (6.1–7.8)	7 (6.4–8.4)	0.02
HbA1c, median (IQR), mmol/mol	53 (43–68)	50 (44–63)	61 (48–74)	54 (43–62)	53 (46–68)	0.02
FPG, median (IQR), mmol/L	7.4 (5.5–9.6)	6.6 (5.1–8.3)	7.4 (5.9–9.3)	7.7 (6–10.1)	7.3 (5.5–10.9)	0.04
Hemoglobin, mean (SD), g/L	119.6 (27.3)	119.7 (26.5)	123.8 (29.6)	122.2 (27.4)	112.1 (24.6)	0.04
Serum albumin, mean (SD), g/L	33.6 (7.9)	34.1 (7.7)	35.2 (8.5)	34 (7.9)	30.9 (7)	<0.01
Total bilirubin, median (IQR), µmol/L	7.0 (5.6–10.4)	7.0 (5.7–10.3)	7.9 (6.3–10.1)	7.2 (5.9–12.6)	5.8 (4.6–9.3)	<0.01
Direct bilirubin, median (IQR), µmol/L	2.1 (1.4–3.2)	2.2 (1.4–3.3)	2.5 (1.7–3.2)	2.0 (1.4–3.7)	1.9 (1.2–2.8)	0.18
CKD stage, stage 1/2/3/4, *n* (%)*	86/63/113/37	25/12/30/5	27/19/24/8	19/19/27/7	15/13/32/17	0.05
BUN, median (IQR), mmol/L	7.8 (6.0–11.0)	7.1 (5.9–9.4)	7.1 (5.4–10.2)	7.6 (5.8–10.1)	9.4 (7.5–14.3)	<0.001
eGFR, median (IQR), mL/min/1.73 m^2^	59.8 (42.9–93.0)	77.4 (46.2–97.5)	60.5 (47.6–97.4)	60.9 (43.2–92.9)	49.0 (34–81.1)	<0.01
24-h Proteinuria, median (IQR), g/d	4.33 (1.99–7.50)	2.81 (1.25–5.52)	4.11 (1.76–6.34)	4.85 (2.53–7.08)	6.19 (3.7–9.68)	<0.001
UA, mean (SD), µmol/L	387.3 (85.0)	390.2 (87.9)	388.7 (81.2)	391.3 (92.4)	379.5 (79.9)	0.82
Triglyceride, median (IQR), mmol/L	1.8 (1.2–2.4)	1.8 (1.3–2.2)	1.8 (1.3–2.3)	1.7 (1.3–2.8)	1.6 (1.2–2.3)	0.53
Cholesterol, median (IQR), mmol/L	5.0 (4.3–6.1)	5.1 (4.3–5.9)	4.8 (4.1–5.8)	5.1 (4.5–6.2)	5 (4.4–6.5)	0.41
HDL, median (IQR), mmol/L	1.3 (1.0–1.6)	1.2 (1–1.5)	1.2 (1–1.6)	1.3 (1–1.6)	1.4 (1.1–1.9)	0.08
LDL, median (IQR), mmol/L	2.9 (2.3–3.8)	2.9 (2.3–3.6)	2.7 (2.1–3.7)	3 (2.4–3.8)	2.8 (2.3–3.8)	0.47
ALT, median (IQR), IU/L	21 (15–29)	19 (14–25)	20 (15–28)	21 (14–31)	24 (17–39)	0.02
AST, median (IQR), IU/L	22 (18–31)	20 (16–26)	23 (18–29)	22 (18–33)	26 (20–36)	0.01
Ca, median (IQR), mmol/L	2.1 (2.0–2.2)	2.2 (2–2.3)	2.1 (2–2.3)	2.1 (2–2.2)	2.1 (2–2.2)	0.58
PO_4_, median (IQR), mmol/L	1.2 (1.1–1.4)	1.2 (1–1.4)	1.2 (1–1.4)	1.2 (1.1–1.4)	1.2 (1.1–1.4)	0.67
Ca × PO_4_, median (IQR), mmol/L	2.6 (2.2–2.9)	2.6 (2.2–2.9)	2.6 (2.2–2.9)	2.6 (2.2–3)	2.4 (2.2–2.9)	0.70
GGT, median (IQR), IU/L	26 (16–49)	20 (12–26)	24 (15–38)	28 (18–52)	46 (19–86)	<0.001
PTH, median (IQR), pg/mL	62.7 (43.7–95.0)	50.4 (38.0–77.9)	65.6 (46.6–111.2)	62.7 (49.4–95.0)	63.7 (47.5–114.0)	0.16
RAAS inhibitors, *n* (%)	239 (79.9)	64 (88.9)	67 (85.9)	61 (84.7)	47 (61.0)	<0.001
Calcitriol use, *n* (%)	88 (29)	20 (28)	24 (31)	23 (32)	21 (27)	0.34
**Renal pathological parameters**						
RPS glomerular classification†, *n* (%)						0.15
Class I	16 (5.4)	4 (5.6)	4 (5.1)	4 (5.6)	4 (5.2)	
Class IIa	58 (19.4)	15 (20.8)	20 (25.6)	17 (23.6)	6 (7.8)	
Class IIb	32 (10.7)	12 (16.7)	7 (9.0)	5 (6.9)	8 (10.4)	
Class III	146 (48.8)	34 (47.2)	36 (46.2)	33 (45.8)	43 (55.8)	
Class IV	47 (15.7)	7 (9.7)	11 (14.1)	13 (18.1)	16 (20.8)	
IFTA†, *n*(%)						<0.001
0	12 (4.0)	3 (4.2)	4 (5.1)	3 (4.2)	2 (2.6)	
1	157 (52.5)	45 (62.5)	49 (62.8)	32 (44.4)	31 (40.3)	
2	100 (33.4)	21 (29.2)	21 (26.9)	28 (38.9)	30 (39.0)	
3	30 (10.0)	3 (4.2)	4 (5.1)	9 (12.5)	14 (18.2)	
Interstitial inflammation†, *n* (%)						0.35
0	11 (3.7)	1 (1.4)	5 (6.4)	4 (5.6)	1 (1.3)	
1	224 (74.9)	58 (80.6)	55 (70.5)	55 (76.4)	56 (72.7)	
2	64 (21.4)	13 (18.1)	18 (23.1)	13 (18.1)	20 (26.0)	
Tubular epithelial degeneration, *n* (%)						0.02
1	30 (10.0)	7 (9.7)	10 (12.8)	10 (13.9)	3 (3.9)	
2	219 (73.2)	59 (81.9)	59 (75.6)	46 (63.9)	55 (71.4)	
3	50 (16.7)	6 (8.3)	9 (11.5)	16 (22.2)	19 (24.7)	
Arteriosclerosis†, *n* (%)						0.08
0	45 (15.1)	17 (23.6)	12 (15.4)	11 (15.3)	5 (6.5)	
1	138 (46.2)	26 (36.1)	40 (51.3)	36 (50.0)	36 (46.8)	
2	111 (37.1)	26 (36.1)	26 (33.3)	25 (34.7)	34 (44.2)	
Arteriolar hyalinosis†, *n* (%)						0.11
0	30 (10.0)	12 (16.7)	10 (12.8)	4 (5.6)	4 (5.2)	
1	80 (26.8)	18 (25.0)	23 (29.5)	21 (29.2)	18 (23.4)	
2	179 (59.9)	37 (51.4)	42 (53.8)	47 (65.3)	53 (68.8)	

Data are presented as the mean (standard) for continuous variables with symmetric distribution, median (25–75th percentiles) for continuous variables with asymmetric distribution, or percentages for categorical variables. Data are shown as number (percentage) of patients for categorical variables. *CKD stage1: eGFR ≥ 90 mL/min/1.73 m^2^; stage 2: eGFR 60–89 mL/min/1.73 m^2^; stage 3: eGFR 30–59 mL/min/1.73 m^2^; stage 4: eGFR 15–29 mL/min/1.73 m^2^. †Defined by RPS diabetic nephropathy classification. IFTA score of 0: no IFTA, score of 1: less than 25% IFTA is present, score of 2: at least 25% but less than 50% of the biopsy has IFTA, score of 3: at least 50% IFTA is present. Tubular epithelial degeneration score of 1: light change, score of 2: mild change, score of 3: severe change. Interstitial inflammation score of 0: interstitial infiltrates are absent, score of 1: interstitial infiltrates only occur around atrophic tubules, score of 2: interstitial infiltrates also in other areas than around atrophic tubules. Arteriosclerosis score of 0: no intimal thickening is present, score of 1: intimal thickening is less than the thickness of the media, score of 2: intimal thickening is more than the thickness of the media. Arteriolar hyalinosis score of 0: no arteriolar hyalinosis is present, score of 1: one arteriole with hyalinosis is present, score of 2: more than one arteriole is observed. ALP: alkaline phosphatase; DM: diabetes mellitus; DR: diabetic retinopathy; CKD: chronic kidney disease; CVD: cardiovascular disease; BMI: body mass index; SBP: systolic blood pressure; DBP: diastolic blood pressure; MAP: mean blood pressure; HbA1c: hemoglobin A1c; FPG: fasting plasma glucose; BUN: blood urea nitrogen; eGFR: estimated glomerular filtration rate; UA: uric acid; HDL: high-density lipoprotein cholesterol; LDL: low-density lipoprotein cholesterol; Ca: calcium; PO_4_: phosphorus; PTH: parathyroid hormone; ALT: alanine transaminase; AST: aspartate aminotransferase; GGT: gamma-glutamyl transferase; RAAS: renin-angiotensin-aldosterone system; RPS: Renal Pathology Society; IFTA: interstitial fibrosis and tubular atrophy.

The baseline pathological characteristics of patients based on the RPS classification system [26] are shown in Table 1. The 299 patients included 16 (5.4%) of class I, 58 (19.4%) of class IIa, 32 (10.7%) of class IIb, 146 (48.8%) of class III, and 47 (15.7%) of class IV. The interstitial fibrosis and tubular atrophy (IFTA) scores were 0 in 12 patients (4.0%), 1 in 157 patients (52.5%), 2 in 100 patients (33.4%), and 3 in 30 patients (10.0%). Glomerular classes were distributed similarly among patients in Q1–Q4. However, IFTA and tubular epithelial degeneration were more severe in patients with higher serum ALP levels. There were no significant differences in interstitial inflammation, atherosclerosis, or arteriolar hyalinosis scores among the patients in the four groups.

### 3.2. Relationship between serum ALP and clinical or renal pathological parameters

In the full cohort of DN patients, the baseline ALP level was significantly, positively associated with 24-h proteinuria (*R^2^* = 0.048, standard *β* = 0.20, *p* = 0.001) but negatively associated with the eGFR (*R^2^* = 0.03, standard *β* = −0.17, *p* < 0.01). In addition, the baseline ALP was positively associated with the GGT (*R^2^* = 0.07, standard *β* = 0.27, *p* < 0.001) and negatively associated with serum albumin (*R^2^* = 0.04, standard *β* =  −0.20, *p*<.001). As for pathological findings, serum ALP was positively associated with IFTA percentages (*R* = 0.04, standard *β* = 0.20, *p* = 0.001). None of the other pathological parameters investigated were significantly associated with serum ALP (*p* > 0.05). The Cochran-Armitage trend test further confirmed that serum ALP was significantly associated with IFTA scores (*p* < 0.001) but was not significantly associated with RPS glomerular classifications (*p* = 0.15).

After adjusting for age, sex, hemoglobin, albumin, and pathological parameters using multivariate regression models, the serum ALP was negatively associated with the eGFR (standard *β* = −0.20, *p* = 0.02), hemoglobin (standard *β* = 0.16, *p* = 0.04), and albumin (standard *β* = −0.18, *p* = 0.02) (Table 2).

**Table 2.** Linear regression analysis between clinical, pathological parameters and serum ALP in patients with type 2 diabetes mellitus and diabetic nephropathy.

	Linear regression analysis		Multivariable regression analysis^a^	
Variable	Standard *β*	*p* Value	Standard *β*	*p* Value
Serum albumin, g/L	−0.20	<0.001	−0.17	0.02
eGFR, mL/min/1.73 m^2^	−0.17	<0.01	−0.19	0.02
24-h Proteinuria, g/d	0.20	<0.01	0.06	0.45
RPS glomerular classification	0.12	<0.01	−0.02	0.78
IFTA	0.20	<0.01	0.10	0.17
Interstitial inflammation	0.05	0.39	−0.08	0.28
Tubular epithelial degeneration	0.11	0.05	0.04	0.58
Arteriosclerosis	0.08	0.17	0.01	0.93
Arteriolar hyalinosis	0.10	0.08	0.04	0.50

^a^Multivariable regression analysis was adjusted for age, sex, serum albumin, hemoglobin, eGFR, proteinuria, and renal pathological parameters (including RPS glomerular classification, IFTA, interstitial inflammation, tubular epithelial degeneration, arteriosclerosis, and arteriolar hyalinosis).

eGFR: estimated glomerular filtration rate; RPS: Renal Pathology Society; IFTA: interstitial fibrosis and tubular atrophy.

### 3.3. Relationship between serum ALP and renal outcome

**Figure 2.**
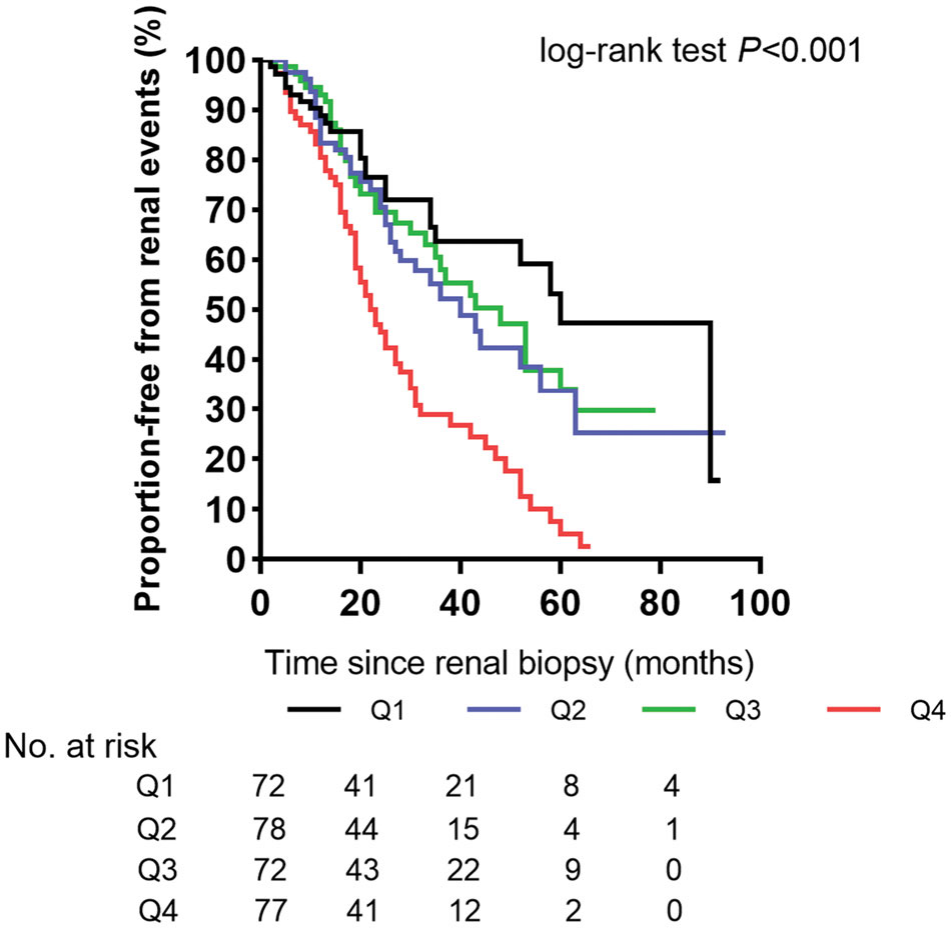
Kaplan–Meier survival curves for renal outcome according to serum alkaline phosphatase levels in the total 299 diabetic patients. Q1–Q4: Quartiles of alkaline phosphatase.

During the follow-up period, ESRD or a 50% decline in the eGFR occurred in 156 (52.2%) patients. The percentages of patients in the Q1, Q2, Q3, and Q4 groups, who progressed to ESRD or a 50% decline in the eGFR were 34.7, 46.2, 47.2, and 79.2%, respectively. The survival curves, depicting the time to renal outcome, showed that the renal survival rates were degraded with increasing serum ALP levels (Figure 2). Univariate Cox analysis showed that the serum ALP level affected the renal survival in patients with T2DM (HR per 1 SD of natural log-transformed serum ALP, 1.24, 95% CI 1.06–1.46, *p<*0.01) (Table 3). After adjusting for age, sex, baseline eGFR, proteinuria, total bilirubin, GGT, and all renal pathological parameters, the highest quartile of ALP (97 IU/L) was significantly associated with renal outcome (HR 2.28, 95% CI 1.38–3.76) compared with the lowest quartile of ALP (≤64 IU/L). When data were further adjusted for the baseline PTH and the usage of the renin-angiotensin-aldosterone system inhibitor, the highest quartile of ALP remained significantly associated with renal outcome (HR 2.38, 95% CI 1.09–5.17) compared with the lowest quartile of ALP (Table 3). Significant interactions were absent for serum ALP quartiles and proteinuria for progression to ESRD or half reduction in eGFR (Supplementary Table 1). When expressed as continuous variable, for each 1-standard deviation increase in the natural log-transformed ALP, there was an associated 25% increased risk for renal outcome (Table 3).

**Table 3.** Association between serum alkaline phosphatase and renal outcome in 299 diabetic patients.

		Survival from ESRD or 50% reduction in the eGFR			
	Hazard ratio (95% Confidence Interval) & P for trend^a^				
		Serum ALP (U/L)			
	Per 1SD ln ALP	Q1 (*n* = 72)	Q2 (*n* = 78)	Q3 (*n* = 72)	Q4 (*n* = 77)
		≤64 IU/L	65–80 IU/L	81–96 IU/L	≥97 IU/L
Unadjusted model	1.26 (1.11–1.43)	1 (reference)	1.52 (0.90–2.56)	1.36 (0.80–2.30)	3.15 (1.95–5.00)
	<0.001		0.11	0.25	<0.001
Model 1#	1.22 (1.04–1.42)	1 (reference)	1.65 (0.97–2.79)	1.48 (0.86–2.54)	2.23 (1.36–3.66)
	0.01		0.06	0.15	<0.01
Model 2§	1.24 (1.06–1.46)	1 (reference)	1.65 (0.97–2.81)	1.56 (0.90–2.69)	2.28 (1.38–3.76)
	<0.01		0.06	0.11	<0.01
Model 3ǂ	1.25 (1.01–1.52)	1 (reference)	2.14 (0.94–4.83)	2.19 (0.9–5.3)	2.38 (1.09–5.17)
	0.04		0.07	0.08	0.03

#Adjusted for age, sex, baseline estimated glomerular filtration rate, proteinuria, gamma-glutamyl transferase, and total bilirubin. §Adjusted for the covariates in model 1 plus renal pathological findings (including the Renal Pathology Society glomerular class, interstitial fibrosis and tubular atrophy, interstitial inflammation, tubular epithelial degeneration, arteriosclerosis, arteriolar hyalinosis). ǂAdjusted for the covariates in model 2 plus parathyroid hormone and the usage of renin-angiotensin-aldosterone system inhibitor. ^a^Linear trend across the quartiles using the median ALP value of each quartile. ALP: alkaline phosphatase; SD: standard deviation; HR: hazard ratio; CI: confidence interval; Q1–Q4: quartiles of alkaline phosphatase.

Analysis of the incremental prognostic value of serum ALP for predicting the risks of renal outcome was performed. The model including serum ALP had a lower AIC than the model that only contained clinical and pathological parameters, which indicates that the former model is superior. Because baseline serum PTH was missing for 149 patients, we performed a sensitivity analysis to investigate the prognostic value of serum ALP in the remaining 150 patients who had PTH data. The incremental prognostic value of serum ALP was more pronounced in patients with PTH data than in the whole cohort (Supplementary Table 2).

### 3.4. Relationship between serum ALP and renal outcome stratified by proteinuria

As proteinuria increased in parallel with serum ALP levels, all patients were stratified into two subgroups according to baseline proteinuria: nephrotic-range proteinuria (24-h proteinuria ≥3.5 g/d) and non-nephrotic-range proteinuria (24-h proteinuria <3.5 g/d).

ESRD or 50% decline in the eGFR occurred in 122 (71.8%) of the 178 patients with nephrotic-range proteinuria. Kaplan–Meier curves, depicting the time to renal outcome, showed that the renal survival rates were exacerbated by the quartiles of serum ALP level (Supplementary Figure 1(a)). Multivariate Cox proportional hazard analysis showed that higher ALP levels were incrementally associated with higher incidence of renal events. Compared with patients in the Q1 group (ALP ≤65 IU/L), the adjusted HRs for renal outcome were 2.84 (95% CI 1.13–5.12) for patients in Q2 group (ALP 66–83 IU/L), 2.94 (95% CI 1.14–5.60) for patients in the Q3 group (ALP 84–102 IU/L) and 3.52 (95% CI 1.82–6.01) for patients in the Q4 group (ALP ≥ 103 IU/L). In the multivariate-adjusted analysis, for each 1-standard deviation increase in the natural log-transformed ALP, there was an associated 40% increased risk for renal outcome (Supplementary Table 3).

In 121 patients with non-nephrotic-range proteinuria, ESRD or 50% reduction in the eGFR occurred in 44 (28.2%) patients. Renal survival rates were not significantly different among the four subgroups according to the quartiles of serum ALP levels (Supplementary Figure 1(b)). By multivariate Cox proportional-hazards analysis, the serum ALP levels were not significantly associated with renal outcome. A significant association between the natural log-transformed ALP and renal outcome was absent as well (Supplementary Table 4).

## 4. Discussion

The present study demonstrated that baseline serum ALP level was negatively correlated with the eGFR and positively associated with 24-h proteinuria in type 2 diabetes patients. Patients with serum ALP ≥ 97 IU/L had a 138% higher risk for ESRD or 50% reduction in the eGFR than those with ALP ≤ 64 IU/L, after adjusting for sociodemographics, baseline eGFR, proteinuria, liver function parameters, PTH, medication administration, and renal pathological findings. In patients with nephrotic-range proteinuria, there was a graded increase in the risk for ESRD with higher ALP quartiles (Q2, Q3, Q4) compared to the reference quartile (Q1) in the multivariate-adjusted analysis. These results indicate that serum ALP might be a novel, noninvasive index for renal outcome in patients with T2DM and DN.

Elevated ALP level was associated with 27 ∼ 65% higher risks for mortality and hospitalization in the general population and survivors of myocardial infarction [33,34]. Epidemiologic studies have shown there is an association between higher ALP levels and mortality in non-dialysis-dependent chronic kidney disease, peritoneal dialysis patients with residual renal function, and incident dialysis patients [9,12,35,36]. High ALP levels were also associated with poor renal function and the progression of chronic kidney disease in the early stages of type 1 diabetes mellitus [20,21]. However, none of these studies investigated the serum ALP-ESRD association in patients with T2DM and DN. We therefore carried out a retrospective observational study in biopsy-proven DN patients with T2DM in our center and for the first time show an association between serum ALP level and ESRD or 50% reduction in the eGFR in biopsy-proven DN patients with T2DM and nephrotic-range proteinuria.

Circulating ALP degrades pyrophosphate, an endogenous anti-calcification factor in the arterial wall [37,38]. Thus, high levels of ALP promot arterial calcification and lead to cardiovascular disease [39]. Increased arterial stiffness led to elevated systemic blood pressure in the defective glomerular capillaries, with low resistance, and exacerbated intraglomerular hypertension and hyperfiltration, and eventually, nephrosclerosis [12,40,41]. Thus, the ALP-ESRD association identified in this study supports the role of arterial calcification in the progression of kidney disease [42].

### 4.1. Kidney disease

Improving Global Outcomes (KDIGO) guidelines recommends monitoring chronic kidney disease mineral and bone disorder biochemical markers, including PTH, calcium, phosphorus, and ALP, in patients with moderate-to-severe chronic kidney disease [43]. Several studies have shown that serum ALP to be a more consistent predictor of adverse outcomes than PTH [6,44]. In kidney transplant patients, elevated pre-transplant ALP levels, but not PTH levels, were associated with increased post-transplant mortality [44]. In our cohort, the relationship between serum ALP and renal outcome was independent of serum PTH levels. The sensitivity analysis performed after excluding patients with missing PTH data produced results that were very similar to those obtained before the exclusion (Supplementary Table 2). Secondary hyperparathyroidism and calcitriol deficiency are the potential causes of increased ALP levels [39]. The response to calcitriol treatment in these patients was largely dependent on the serum PTH levels. A recent meta-analysis, however, showed that activated vitamin D supplements decreased the serum ALP level more effectively than lowering the PTH levels [45,46]. Thus, when patients are treated with calcitriol, addjusting the combination of serum PTH and ALP levels might be a good strategy to help physicians and guide the reduction or discontinuation of calcitriol usage.

ALP is normally considered as a pathogenic player in abnormal mineralization hemostasis; it participates in collagen calcification during bone formation [47] and it is also a matrix-modifying player. Our study discovered that ALP was positively associated with interstitial fibrosis in patients with DN. Accumulating studies have shown that serum ALP is a fibrosis marker in chronic interstitial disorders [48–50]. The underlying mechanisms of ALP-induced fibrosis might be via the Wnt-dependent pathway by secreted Frizzled-related protein 2 (sFRP2) [51]. sFRP2 regulates extracellular matrix remodeling by the activation of Wnt-signaling. Overactivation of Wnt signaling has been shown to induce fibroblast proliferation, myofibroblastic trans-differentiation, and renal fibrosis [52,53]. In a congenital obstructive uropathy model, sFRP2 expression was increased in obstructed kidneys with fibrosis [54]. Therefore, we hypothesized that ALP might play a role in kidney fibrosis through the sFRP2-Wnt signaling pathway.

Although serum ALP was not associated with glomerular injury, there was a cross-sectional association between serum ALP and baseline proteinuria. This result was consistent with previous studies [9,12]. Proteinuria can be glomerular, resulting from an impairment of the glomerular filtration apparatus, or tubular from diminished tubular resorption of low-molecular-weight proteins [55]. In DN, glomerular proteinuria places a large burden on the tubular epithelial cells, and the damage to these cells becomes the main driver of disease progression [56]. In physiological conditions, tubular cells express ALP at a higher level than glomerular endothelial or mesangial cells [57,58]. Thus, the strong correlation between serum ALP and proteinuria highlighted that the proximal tubules play a significant role in determining the magnitude of proteinuria [59]. The ALP released from tubular cells damaged by glomerular hyperfiltration could contribute to increased levels of urinary ALP in patients with DN [57,60].

The negative association between serum ALP and renal outcome in patients with non-nephrotic range proteinuria could have resulted from insufficient power in our study due to the limited number of patients and low incidence of renal events. The absence of interaction between serum ALP and proteinuria suggests that serum ALP increased the risk of progression to ESRD or 50% reduction in the eGFR in patients with nephrotic-range proteinuria.

This study has several limitations. Due to the retrospective analysis design, PTH was missing in 49% of patients in this cohort. However, the relationship between serum ALP and renal outcome did not change before and after PTH adjustment in the multivariate Cox proportional-hazard models. Also, it was difficult to identify patients with active liver disease, which may influence the ALP levels. However, multivariate Cox analysis was performed, adjusting for liver function parameters, to reduce such confounding.

In summary, this study revealed that higher serum ALP was associated with ESRD in patients with T2DM and massive proteinuria. Also, our findings highlight an urgent need attention to ALP levels in kidney fibrosis in DN. The potential therapeutic interventions targeting ALP to alleviate DN progression and fibrosis warrant further investigation.

## Supplementary Material

Supplemental Material

## Data Availability

The datasets generated for this study are available on request to the corresponding author.
