# Supplementary material for: Association between serum alkaline phosphatase and renal outcome in patients with type 2 diabetes mellitus
Source: Ren Fail. 2020 Aug 12;42(1):818–28. doi: 10.1080/0886022X.2020.1804402 (PMC7472471; doi:10.1080/0886022X.2020.1804402)
Supplement: Supplemental Material [file IRNF_A_1804402_SM8396.pdf]

Supplementary Table 1. The *P* for interaction between serum ALP and proteinuria in the total 299 type 2 diabetic patients with diabetic nephropathy.

	Survival from ESRD or 50% reduction in the eGFR	
	Adjusted hazard ratio (95% Confidence Interval) #	$P_{interaction}$ with proteinuria
ALP		
Q1 (≤64 IU/L)	1 (reference)	
Q2 (65–80 IU/L)	1.06 (0.94-1.19)	0.37
Q3 (81–96 IU/L)	0.96 (0.85-1.08)	0.51
Q4 (≥97 IU/L)	0.97 (0.88-1.08)	0.61

#Adjusted for age, sex, baseline estimated glomerular filtration rate and proteinuria, gamma-glutamyl transferase, total bilirubin, and renal pathological findings (including the Renal Pathology Society glomerular class, interstitial fibrosis and tubular atrophy, interstitial inflammation, tubular epithelial degeneration, arteriosclerosis, arteriolar hyalinosis). **Abbreviation:** ALP, alkaline phosphatase; ESRD, end-stage renal disease; eGFR, estimated glomerular filtration rate; Q1–Q4, Quartiles of alkaline phosphatase.

Supplementary Table 2. The incremental prognostic value of serum ALP for predicting the risk of renal outcome.

Statistics	Total 299 patients		150 patients who had PTH data	
	Model 1 [a]	Model 1 + ALP	Model 1 [a]	Model 1 + ALP
Harrell's C Concordance statistics	0.751	0.759	0.762	0.778*
LR $\chi 2$	115.8	121.1	117.1	122.4*
AIC	1391.9	1388.5	890.9	738.7

[a] Model 1 included estimated glomerular filtration rate, urinary protein, and Renal Pathological Society pathological parameters. LR χ^2, likelihood ratio Chi–square statistics; AIC, Akaike information criterion. *, P＜0.05 (versus Model 1).

Supplementary Table 3. Univariate and multivariable Cox models for time to renal outcome in type 2 diabetic patients with nephrotic-range proteinuria.

	Survival from ESRD or 50% reduction in the eGFR				
	Per 1SD	Hazard ratio (95% Confidence Interval) & P for trend [a] Serum ALP (U/L)			
	ln ALP	Q1 (n = 46) ⩽65 IU/L	Q2 (n = 49) 66–83 IU/L	Q3 (n = 42) 84–102 IU/L	Q4 (n = 41) ⩾103 IU/L
Unadjusted model	1.49 (1.25–1.78)	1 (reference)	2.06 (1.09–3.90)	2.81 (1.51–5.24)	3.48 (1.91–6.35)
	< 0.001		0.03	<0.01	<0.001
Model 1 #	1.24 (1.02–1.51)	1 (reference)	2.13 (1.12–4.05)	2.46 (1.30–4.68)	2.50 (1.35–4.63)
	0.03		0.02	0.01	< 0.01
Model 2 §	1.35 (1.09–1.66)	1 (reference)	2.22 (1.16–4.25)	2.64 (1.38–5.08)	2.96 (1.56–5.60)
	< 0.01		0.02	<0.01	<0.001
Model 3ǂ	1.40 (1.10–1.70)	1 (reference)	2.84 (1.13－5.12)	2.94 (1.14－5.60)	3.52 (1.82－6.01)
	0.04		0.03	0.03	0.01

Model 1#, adjusted for age, sex, baseline estimated glomerular filtration rate, and proteinuria, gamma-glutamyl transferase and total bilirubin. Model 2 §, adjusted for covariates in model 1 plus renal pathological findings (including the Renal Pathology Society glomerular class, interstitial fibrosis and tubular atrophy, interstitial inflammation, tubular epithelial degeneration, arteriosclerosis, arteriolar hyalinosis). Model 3 ǂ, adjusted for the covariates in model 2 plus parathyroid hormone and the usage of renin-angiotensin-aldosterone system inhibitor. [a] Linear trend across the quartiles using the median ALP value of each quartile. **Abbreviation:** ALP, alkaline phosphatase; SD, standard deviation; HR, hazard ratio; CI, confidence interval; Q1–Q4, Quartiles of alkaline phosphatase.

Supplementary Table 4. Univariate and multivariable Cox models for time to renal outcome in type 2 diabetic patients with non-nephrotic-range proteinuria.

	Survival from ESRD or 50% reduction in the eGFR				
		Hazard ratio (95% Confidence Interval) & P for trend [a]			
	Per 1SD	Serum ALP (U/L)			
	ln ALP	Q1 (n = 30) ≤63 IU/L	Q2 (n = 31) 64–75 IU/L	Q3 (n = 30) 76–87 IU/L	Q4 (n = 30) ≥88 IU/L
Unadjusted model	1.06 (0.81–1.39)	1 (reference)	2.73 (1.03–7.21)	1.49 (0.54–4.1)	2.87 (1.1–7.49)
	0.67		0.04	0.44	0.03
Model 1 #	1.21 (0.80–1.84)	1 (reference)	1.7 (0.62–4.72)	1.43 (0.49–4.14)	2.64 (0.93–7.47)
	0.37		0.3	0.51	0.07
Model 2 §	0.99 (0.63–1.55)	1 (reference)	2.11 (0.76–5.86)	1.18 (0.40–3.47)	1.83 (0.61–5.47)
	0.97		0.15	0.76	0.28
Model 3ǂ	1.05 (0.64–1.84)	1 (reference)	1.95 (0.58－3.97)	1.17 (0.47－3.71)	1.80 (0.60－4.28)
	0.51		0.13	0.14	0.12

Model 1 #, adjusted for age, sex, baseline estimated glomerular filtration rate, and proteinuria, gamma-glutamyl transferase and total bilirubin. Model 2 §, adjusted for covariates in model 1 plus renal pathological findings (including the Renal Pathology Society glomerular class, interstitial fibrosis and tubular atrophy, interstitial inflammation, tubular epithelial degeneration, arteriosclerosis, arteriolar hyalinosis). Model 3 ǂ, adjusted for the covariates in model 2 plus parathyroid hormone and the usage of renin-angiotensin-aldosterone system inhibitor. [a] Linear trend across the quartiles using the median ALP value of each quartile. **Abbreviation:** ALP, alkaline phosphatase; SD, standard deviation; HR, hazard ratio; CI, confidence interval; Q1–Q4, Quartiles of alkaline phosphatase.

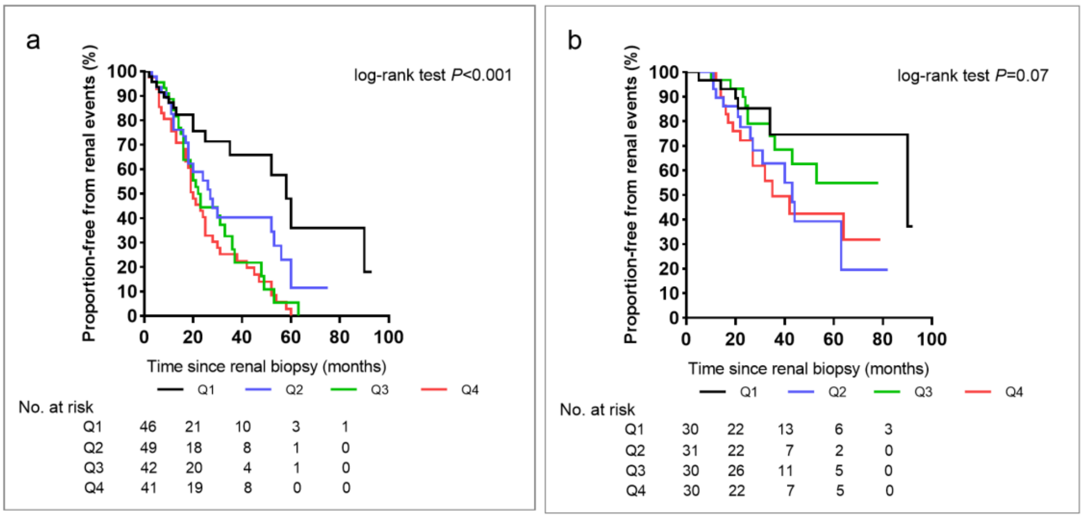

Supplementary Figure 1. Kaplan-Meier survival curves for renal outcome according to serum alkaline phosphatase levels in patients with nephrotic-range proteinuria (a), or in patients with non-nephrotic-range proteinuria (b). Q1–Q4, Quartiles of alkaline phosphatase.
